# Studying the Relationship between the Antiviral Activity and the Structure of ἰ-Carrageenan Using Ultrasonication

**DOI:** 10.3390/ijms241814200

**Published:** 2023-09-17

**Authors:** Oshrat Levy-Ontman, Eiman Abu-Galiyun, Mahmoud Huleihel

**Affiliations:** 1 Department of Chemical and Green Engineering, Shamoon College of Engineering, Beer-Sheva 8410802, Israel; 2 Department of Microbiology, Immunology and Genetics, Faculty of Health Sciences, Ben-Gurion University of the Negev, Beer-Sheva 8410501, Israel; mahmoudh@bgu.ac.il

**Keywords:** antiviral, carrageenan, herpesvirus, physicochemical, ultrasonication

## Abstract

ἰ-carrageenan is a linear macroalgal polysaccharide that is well known for its antiviral bioactivity. Although it is considered a candidate for antiviral therapeutics, its application is highly limited due to its low solubility and high viscosity, which lower its adsorption efficiency. With the aim of deriving an active ἰ-carrageenan fragment with an improved adsorption capacity, we studied the effects of ultrasonication on structural changes in ἰ-carrageenan with respect to changes in its bioactivity against herpesviruses. An FTIR analysis revealed that ultrasonication increased the hydrophilicity of ἰ-carrageenan without changing its functional groups, and a rheological analysis demonstrated that it gradually decreased the strength of the polysaccharide gel, which completely lost its gel structure and formed small nanoparticles after 30 min of ultrasonication. Concomitantly with these physicochemical changes, a plaque assay revealed that longer ultrasonication increased the antiviral activity of ἰ-carrageenan against two herpesviruses, namely, HSV-1 and VZV. Finally, we separated the 30-min ultrasonicated ἰ-carrageenan into four fractions and found that fractions with a lower molecular weight were significantly less active against both herpesviruses than those with a higher molecular weight. Our findings show that ultrasonication induces physicochemical changes in ἰ-carrageenan that increase its antiviral bioactivity.

## 1. Introduction

**Figure 1:**
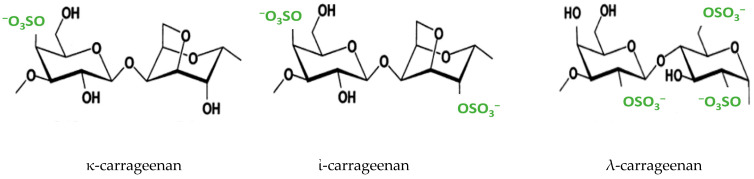
A schematic illustration of carrageenan forms.

Carrageenan is a family of algae-derived sulfated linear and renewable polysaccharides composed of D-galactose units linked alternately with α-1,4 and β-1,3 linkages, modified by 3,6-anhydro bridges, and substituted with ester sulfate groups [1,2]. Members of the carrageenan family have attracted much attention due to the unique physicochemical behavior of these polysaccharides, which are today ubiquitous as gelling and thickening agents in the biomedical and food industries, as agents for catalysis in the cosmetics industry, and in many other applications [2,3,4]. The most well-known forms of carrageenan polysaccharides are iota-, kappa-, and lambda- (ἰ-, κ-, and λ-, respectively) carrageenan, which differ from each other in their sulfation patterns and the presence of 3,6-anhydrogalactopyranose on the D-units [5,6]: κ-carrageenan comprises 25–30% sulfate ester groups at position 4 of the glucose units and 28–35% 3,6-anhydrogalactose; ἰ-carrageenan comprises 28–38% sulfate ester groups at the position 4 of the glucose units and at position 2 of the anhydrogalactopyranose and 25–30% 3,6-anhydrogalactose; and λ-carrageenan lacks 3,6-anhydrogalactose and comprises 32–39% sulfate ester at the 2 and 6 positions of the galactose unit [7]. A schematic illustration of the carrageenan forms is shown in Figure 1.

Carrageenan demonstrates antiviral bioactivity against numerous strains and groups of viruses, including various herpesviruses [8,9], both in vitro (with a half-maximal inhibitory concentration (IC50) of 0.01–34.3 µg/mL) and in vivo (in both mouse and cat models). Its superior safety profile has further accelerated the clinical development of carrageenan as an atopic gel for the prevention of human papillomavirus and the transmission of human immunodeficiency virus and as a nasal spray for a reduction in viral loads in nasal lavages in patients with early symptoms of the common cold [10]. The antiviral bioactivity of carrageenan appears to be correlated with several factors, such as its molecular weight, monosaccharide composition, and sulfate content. Notably, the regiochemistry of the sulfate groups onto the carbohydrate domain has been shown to be crucial for the antiviral activity of carrageenan [11,12].

Like other polysaccharides, the high molecular weight of carrageenan reduces its solubility and increases its viscosity, thereby reducing its ability to penetrate cells. Therefore, an ongoing challenge is to isolate bioactive low molecular weight sites or fractions, which may improve treatment efficacy. The most widespread methods for the degradation of polysaccharides are enzymatic methods, wherein the ability to control the enzyme hydrolysis capacity is limited, and chemical methods, which employ hazardous chemicals [13,14,15]. A preferable alternative is to use ultrasound-assisted polysaccharide degradation [16,17,18,19,20]: an effective, easy-to-use, easily adaptable, and environmentally friendly method of polymer modification [13], which has been shown to change the structure and properties of various polysaccharides [21,22,23,24,25]. However, although ultrasound-assisted techniques can potentially enhance the extraction, modification, and processing of carrageenan to improve its antiviral properties, only a few studies investigated the structural effects of ultrasound on carrageenan, and these studies focused on κ-carrageenan, employed highly specific conditions (temperature, water/polysaccharide ratios, sonication frequency, time of exposure, etc.), and did not investigate the resulting changes in antiviral bioactivity [25,26,27]. We previously reported that ἰ-carrageenan exhibits impressive antiviral activity against varicella zoster virus (VZV) [12], but it is yet unclear how ultrasound affects the physicochemical properties of ἰ-carrageenan and, accordingly, its antiviral activity.

To bridge these gaps, the aims of this study were to elucidate the structural (physical and chemical) changes that ultrasonication produces in ἰ-carrageenan, evaluate its in vitro antiviral activity against herpesviruses, and determine whether the ultrasonication releases fractions with low molecular weight and high antiviral activity, which may then be used as antiviral therapeutic drugs. We focused on the antiviral activity of ἰ-carrageenan against herpesviruses, as these viruses infect the human population at high rates (50–90% of people worldwide) and are involved in a wide variety of diseases that cause symptoms ranging from mild infections to life-threatening and fatal diseases. After the acute infection stage, herpesviruses become latent and persist in different cell types, depending on the type of herpesvirus, for the entire life of the organism. Within the large family of herpesviruses, we focused on two of the most common and well-known viruses—herpes simplex virus-1 (HSV-1) and VZV—which belong to the α-subgroup of herpesviruses and whose latent infection occurs in neuronal cells within the peripheral ganglia of humans [28]. HSV-1 typically results in herpes labialis following the primary infection, but it can also occur in other places in the body (e.g., the genitals) and, in rare cases, can result in encephalitis. VZV usually infects during childhood and causes varicella (chickenpox) and, due to reactivation of the latent virus, produces herpes zoster (shingles) and causes severe pain in the area of the latently infected ganglia. The common treatment for infection by these two herpesviruses is symptomatic and is based mainly on nucleoside analogs, which inhibit the synthesis of the viral DNA. Although these treatments are generally effective, they have undesired side effects, they are ineffective against some treatment-resistant strains, and they are not suitable for treating infections in immunocompromised or immune-deficient patients, highlighting the need for alternative treatment options [29,30].

## 2. Results and Discussion

**Figure 2:**
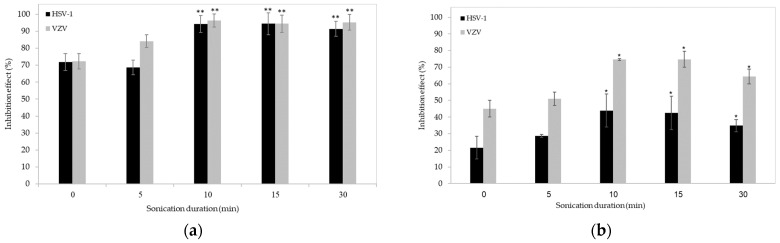
Ultrasonication increases the antiviral activity of 1 µg/mL (**a**) and 0.1 µg/mL (**b**) ἰ-carrageenan against both HSV-1 (black) and VZV (gray). Vero cells were infected with either HSV-1 or VZV (0.01 m.o.i) and treated with the native or ultrasonicated ἰ-carrageenan, both during the infection and 2 days later. Antiviral activity was evaluated using a plaque assay and is shown as mean (±SEM) inhibition, calculated as the number of plaques in the treated cells minus those in the untreated cells, divided by the number of plaques in the untreated cells (n = 5 independent experiments for each condition). * *p* < 0.5 and ** *p* < 0.001 vs. the respective infected, untreated cell (unpaired two-tailed Student’s *t*-test).

First, we determined the effect of ultrasonication duration (5–30 min) on the antiviral activity of ἰ-carrageenan (1.0 µg/mL and 0.1 µg/mL) against HSV-1 and VZV, using a plaque assay in Vero cells (Figure 2). In vitro cytotoxicity measurements (see Materials and Methods for details) showed that the number, morphology, and metabolic activity of the cells were not different between untreated and ultrasonicated preparations (*p* > 0.05), indicating no cytotoxic activity following ultrasonication. However, ultrasonication for 10 min or longer significantly increased the antiviral activity of ἰ-carrageenan against both HSV-1 and VZV, reaching up to 100% inhibition for 1 µg/mL ἰ-carrageenan (Figure 2a) and up to 70% inhibition for 0.1 µg/mL ἰ-carrageenan (Figure 2b) after 10 min of ultrasonication. In addition, the antiviral effect of the 0.1 µg/mL ἰ-carrageenan was significantly greater against VZV than against HSV-1 (*p* < 0.05), regardless of the ultrasonication duration.

**Figure 3:**
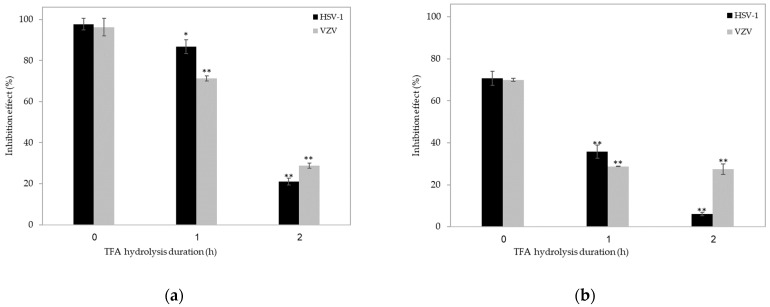
Trifluoroacetic acid (TFA) hydrolysis (1 h or 2 h treatment) decreases the antiviral activity of 1 µg/mL (**a**) and 10 µg/mL (**b**) ἰ-carrageenan against HSV-1 (black) and VZV (gray), as compared with the native ἰ-carrageenan. n = 5 independent experiments for each condition. * *p* < 0.5 and ** *p* < 0.001 vs. the respective infected, untreated cell (unpaired two-tailed Student’s *t*-test). See Figure 1 for experimental details.

Next, we compared the antiviral efficiency of ἰ-carrageenan following ultrasonication to its efficiency following acidic hydrolysis (1 µg/mL or 10 µg/mL ἰ-carrageenan, treated with trifluoroacetic acid (TFA) for 1 h or 2 h), which is a conventional and widely used method for separating fractions from polysaccharides. As in the case of ultrasonication, the acid hydrolysis did not induce cytotoxicity, as the number, morphology, and metabolic activity of the treated cells were not different from those in untreated cells (*p* > 0.05); however, the treatment reduced the antiviral activity of ἰ-carrageenan in a treatment duration-dependent manner (Figure 3). Notably, in κ-carrageenan, mild acid hydrolysis has been shown to generate a mixture of oligosaccharides with heterogeneous chemical structures and different molecular weights [31,32]. Yang et al. [33] reported that mild acid hydrolysis resulted in breaking inside α-1.3 links and in the production of odd-numbered oligosaccharide fragments (three-, penta-, and heptasaccharides), while even-numbered fragments after the breaking of β-1.4-linked oligosaccharides were detected as a minor component. Our findings are in agreement with a previous study [34], which demonstrated that low molecular weight derivatives of various κ-/β-carrageenan forms obtained by acid hydrolysis yielded low antiviral bioactivity against tobacco mosaic virus, as compared with the native carrageenan.

**Figure 4:**
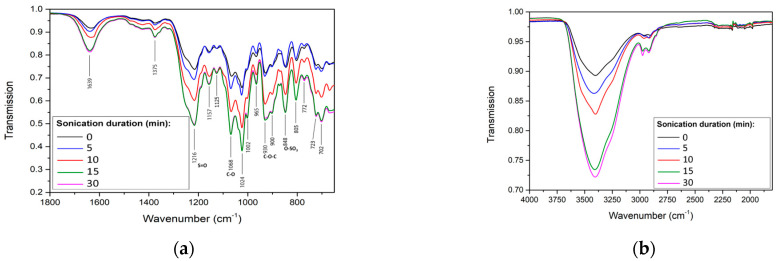
ATR-FTIR spectra of ἰ-carrageenan at the range of 680–1800 cm^−1^ (**a**) and 1850–4000 cm^−1^ (**b**) after 0, 5, 10, 15, or 30 min of ultrasonication.

An ATR-FTIR analysis revealed that ultrasonication (5–30 min) did not considerably change the FTIR spectrum of ἰ-carrageenan (Figure 4), which contained all the expected peaks [35]: a peak at 3200–3400 cm^−1^, resulting from the assigned hydrogen O–H stretching vibrations bonds; a peak at 1068 cm^−1^, resulting from the assigned C–O stretching of 3,6 galactose; peaks at 930 cm^−1^ and 848 cm^−1^, resulting from the assigned C–O–C of 3,6-anhydro-D-galactose and the O–SO_3_ stretching vibration at D-galactose-4-sulfate, respectively; a peak at 805 cm^−1^, resulting from the assigned D-galactose-2-sulfate [36,37]; and a peak at 1216 cm^−1^, assigned to the characteristic band of S=O of sulfate ester (O=S=O symmetric vibration) and indicating that no new covalent bonds had formed. These findings are in line with those of a previous study on κ-carrageenan, which showed that ultrasonication does not change its functional groups and molecular framework [25,26]. Notably, the intensity of the OH stretching band and the O=S=O symmetric vibration of ἰ-carrageenan increased with ultrasonication duration, suggesting that ultrasound facilitates the breaking of the intermolecular interactions between the polysaccharide chains. A similar phenomenon was reported after ultrasonicating κ-carrageenan at an output frequency of 45 kHz [25], where 1, 3, or 5 min of ultrasonication increased the intensity of the OH band. The authors of that study suggested that ultrasonication enhanced the interactions between water and the polysaccharides by intramolecular hydrogen bonding and that it easily affected the intermolecular interactions; as ultrasonication broke the intermolecular interactions, the water molecules were included in the polysaccharide molecules to interact in other ways [25].

**Figure 5:**
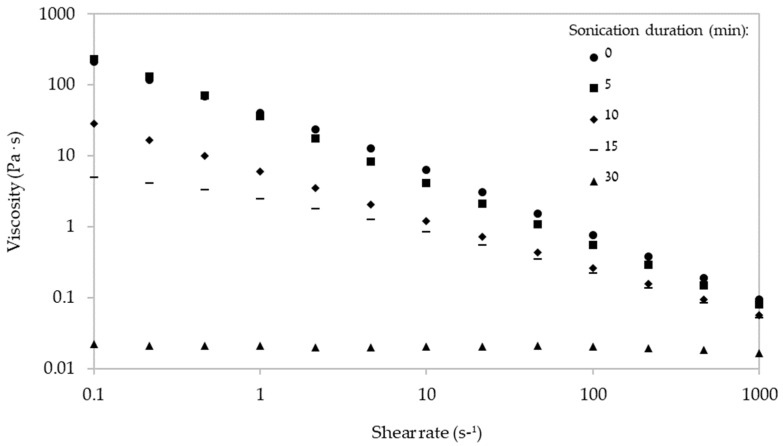
Viscosity of ἰ-carrageenan (1% *w*/*v*) as a function of shear rate after different ultrasonication durations at 25 °C.

Viscosity measurements (Figure 5) revealed that ἰ-carrageenan and its ultrasonicated (up to 15 min) forms displayed a typical non-Newtonian shear-thinning behavior, and that viscosity decreased with increasing ultrasonication duration—possibly due to a decrease in the self-association of the intermolecular interactions between the polysaccharide chains [38].

To gain further insights into the shear-thinning regime of each preparation, we calculated the power law index constant (n) of ἰ-carrageenan after different ultrasonication durations (Table 1). To this end, we fitted the data presented in Figure 5 to the mathematical model of Ostwald-de Waele (power law), which is suitable for shear-thinning fluids and weak gels. As expected, all the ἰ-carrageenan forms that were sonicated for up to 15 min exhibited non-Newtonian behavior (n < 0.5), whereas ultrasonication for 30 min resulted in Newtonian behavior (n~1). The coefficient of determination was high (R^2^ ≥ 0.946) for the fitting of all the examined polysaccharides (Table 1).

**Figure 6:**
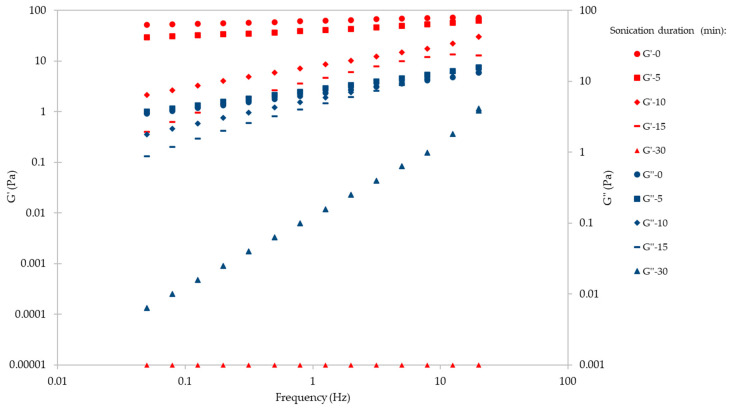
The elastic (G′) and viscoelastic (G″) moduli of ἰ-carrageenan (1% *w*/*v*, 25 °C) after different ultrasonication durations, as a function of angular frequency.

To compare the mechanical spectra of the native and ultrasonicated ἰ-carrageenan forms, the storage (or elastic) modulus (G′) and the viscoelastic (or loss) modulus (G″) were measured as a function of the angular frequency. Except in the sample of the 30 min ultrasonicated ἰ-carrageenan, G′ exceeded G″ in all samples—indicating a gel-like structure—but the stiffness of the gel decreased with increasing ultrasonication duration (Figure 6). In the 30 min ultrasonicated sample, G″ exceeded G′, indicating that ἰ-carrageenan lost its gel-like structure and behaved as a liquid.

The ultrasonication-induced physicochemical changes in ἰ-carrageenan are similar to those reported for κ-carrageenan by Kang et al. [39], who demonstrated that ultrasonication decreased the molecular weight because it changed the tight crystalline structure to random coils, thereby exposing the hydrophilic parts to water. We conclude that ultrasonication affects the texture of ἰ-carrageenan, depending on the duration of treatment.

**Figure 7:**
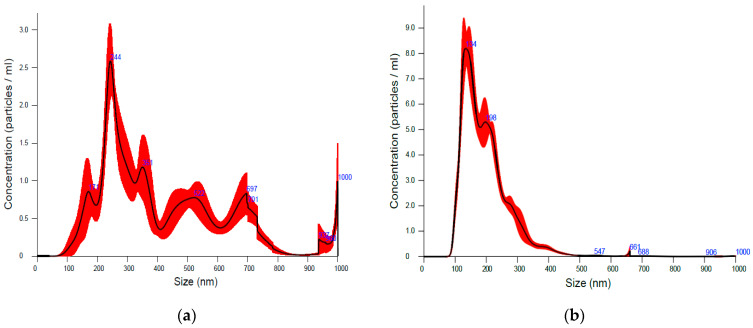
Nanoparticle tracking analysis of native (**a**) and 30 min-ultrasonicated (**b**) ἰ-carrageenan.

To facilitate the use of carrageenan in biomedical applications, extensive efforts have been made to form carrageenan-based nanoparticles, mostly through ionic gelation or by mixing carrageenan with cationic polymers to form polyelectrolyte complexes [40]. As ultrasonication is also known to form nanoparticles, we employed a nanoparticle tracking analysis (Figure 7) and found that the ultrasound-induced cavitation forces resulted in the formation of smaller ἰ-carrageenan fragments in the narrow range of 90–500 nm. The ultrasonicated ἰ-carrageenan that contained these nanoparticles demonstrated a high surface-to-volume ratio and an increased surface functionality—two features that make it a highly promising candidate for drug delivery.

Taken together, our findings suggest that the increased antiviral efficacy of the ultrasonicated ἰ-carrageenan resulted from structural changes in the polysaccharide, such that a longer ultrasonication increased its flexibility and decreased the entanglements between the polysaccharide chains, resulting in more available sulfate groups that better interact with the virus.

Finally, we aimed to obtain antiviral ἰ-carrageenan fractions from the 30 min-ultrasonicated preparation, using herpesvirus as a model target. Comparing the sugar content of the ultrasonicated fraction to that of the native ἰ-carrageenan analogs revealed that only a small part of the polysaccharide was fragmented into <100 kDa fractions (Table 2). These findings support the FTIR analysis and indicate that the ultrasonication did not change the polysaccharide network, although it reduced the intermolecular chain connections. Indeed, the ultrasonication of polysaccharides is beneficial in various industries, such as in food and cosmetics, because it does not significantly affect the molecular weight of the polysaccharide and does not lead to the formation of small fractions of poligeenan, which are known to be toxic [41].

**Figure 8:**
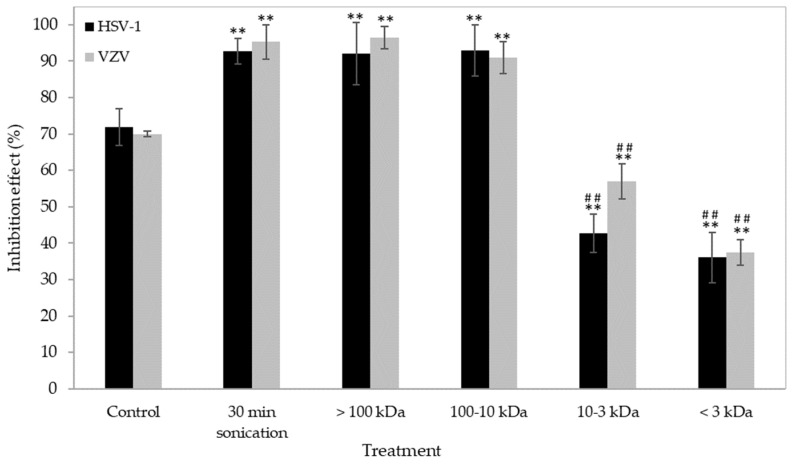
Antiviral activity of fractions of ἰ-carrageenan against HSV-1 and VZV. n = 5 independent experiments for each condition, ** *p* < 0.001 vs. the control (cells treated with the native ἰ-carrageenan), ## *p* < 0.001 vs. the cells treated with the 30 min-ultrasonicated ἰ-carrageenan (unpaired two-tailed Student’s *t*-test). See Figure 1 for experimental details.

The antiviral activity of the ultrasonicated ἰ-carrageenan fractions with low molecular weights (<10 kDa) was less effective against both HSV-1 and VZV than that of the ultrasonicated fractions with higher molecular weights and the native ἰ-carrageenan (Figure 8). Fractions with a higher molecular weight (10–100 kDa and >100 kDa) showed a similar antiviral activity, which was not significantly different from that found in the 30 min-ultrasonicated preparation.

## 3. Materials and Methods

### 3.1. Preparation of ἰ-Carrageenan and Its Ultrasonicated Forms

To prepare the ἰ-carrageenan solution, 1 g of ἰ-carrageenan powder (Sigma-Aldrich, Jerusalem, Israel) was dissolved in 100 mL DDW and mixed for 1 h at 50 °C, yielding a 1% *w*/*v* aqueous solution. For ultrasonication, 15 mL of the solution was ultrasonicated at 20 kHz, 750 W, using a VCX 750 microprocessor (Sonic & Materials, Inc., Newtown, CT, USA) with a standard ½” diameter probe and a 13 mm tip. The solution was ultrasonicated at 50% amplitude, using 30 s pulses and 30 s intervals, for a net ultrasonication duration of 5–30 min.

### 3.2. Acid Hydrolysis

Trifluoroacetic acid (TFA, analytical grade; Sigma-Aldrich) was added to 15 mL of the 1% *w*/*v* ἰ-carrageenan solution to reach a final TFA concentration of 2 M. This solution was subjected to either 1 h or 2 h of mixing with a magnetic stirrer in an oil bath, which was constantly maintained at 60 °C by using a hot plate. After the treatment, the solution was cooled to room temperature, evaporated to dryness with an external N_2_ stream, redissolved in 200 μL of 2-propanol (analytical grade; Sigma-Aldrich), and then evaporated to dryness in a vacuum centrifuge to remove residual TFA. The pellet was redissolved in 15 mL DDW and was used as the TFA-treated preparation.

### 3.3. Fractionation

The native and the 30 min-ultrasonicated ἰ-carrageenan were separated into fractions of different sizes (<3 kDa, 3–10 kDa, 10–100 kDa, and >100 kDa) by dispersing the preparations onto ultracentrifuge filters (Amicon, Sigma-Aldrich, Jerusalem, Israel) and then collecting the fractions according to the user manual. Each fraction was freeze-dried by a lyophilizer and then redissolved in DDW to reach a stock solution with a 1% *w*/*v* sugar content.

### 3.4. Sugar Content Determination

The sugar content of the samples was determined by the phenol-sulfuric method [42]. Briefly, 1 mL of the sample was mixed for a few seconds with 1 mL of 5% phenol and 5 mL of 98% sulfuric acid (both analytical grade; Sigma-Aldrich) and incubated at room temperature. Then, the mixed solution was left in ice for 30 min, and the absorbance of the developed color was determined spectrophotometrically (490 nm). The sugar content was deduced from the absorbance by comparing it with a standard curve (0–100 g/mL) of galactose.

### 3.5. In Vitro Cytotoxicity Measurements in Vero Cells

African green monkey kidney (Vero) cells (American Type Culture Collection, Rockville, MD, USA) were grown in an RPMI medium with 10% fetal calf serum, 1% glutamine, and 50 µg/mL antibiotic mixture (penicillin and streptomycin) and incubated at 37 °C in humidified air containing 5% CO_2_. All the supplements for the Vero cultivation were purchased from Biological Industries (Beit-Haemek, Israel). The cells were treated with various concentrations (up to 100 µg/mL) of ἰ-carrageenan before and after the ultrasonication, TFA hydrolysis, or fractionation treatment, and their cytotoxicity was tested by three methods during a three-day period: (1) counting the cells by a Neubauer hemocytometer as an indication of their replication rate; (2) daily morphological observations using an inverted optical microscope; and (3) the (3-(4,5-dimethylthiazol-2-yl)-2,5-diphenyl tetrazolium bromide (MTT; Sigma-Aldrich) method, as described previously [43]. Untreated cells were used as controls for comparison, such that the fold percentage cytotoxicity effect was calculated as the ratio between the number of cells counted following the treatment divided by the number of untreated cells multiplied by 100.

### 3.6. In Vitro Antiviral Activity Efficacy

The antiviral activity of each treatment (ultrasonication, TFA, or fractionation) against HSV-1 and VZV was evaluated by a plaque assay [44]. Briefly, Vero cells were seeded at 0.2 × 10^6^ cells/well in 24-well culture plates and incubated for 24 h. The medium was removed, and each well was infected for 2 h with 0.01 m.o.i of VZV or HSV-1 at 37 °C. Then, the medium was removed, and the cells were covered with a new layer of medium containing carboxymethylcellulose (CMC) in the presence or absence of the treatment solution for 2 days. Next, the CMC overlay was removed, and the cell monolayers were fixed with 10% formaldehyde in saline, stained with crystal violet, and the plaques counted. The antiviral effect was calculated as the number of plaques in the treated cells minus the number of plaques in the untreated cells, divided by the number of plaques in the untreated cells.

VZV and HSV-1 were obtained from ATCC (VR-1433 and VR-733, respectively). A stock solution of the viruses was prepared by propagating them to >10^6^ plaque-forming units (PFU)/mL in Vero cells, and it was stored at −80 °C.

### 3.7. Statistical Analyses

All experiments were repeated five times for each condition, and data are expressed as the means ± SEM of the five experiments. The statistical analysis was performed with Graph-Pad Prism 7.02 Software (San Diego, CA, USA), and the statistical significance of the differences between the means of various subgroups was assessed by unpaired two-tailed Student’s *t*-test, considering *p* < 0.05 as a statistically significant difference.

### 3.8. Fourier-Transform Infrared (FTIR) Analyses

The FTIR analyses were conducted using a Nicolet 6700 FTIR spectrophotometer (Thermo Scientific, Waltham, MA, USA) with an attenuated total reflectance (ATR) device outfitted with a diamond crystal plate. The recorded spectra were the means of 36 spectra taken in the wavelength range of 650–4000 cm^−1^ with a 0.5 cm^−1^ resolution, and atmospheric correction switched on at room temperature (25 °C).

### 3.9. Rheological Measurements

The viscosity of the native (1% *w*/*v*) and ultrasonicated ἰ-carrageenan solutions was determined by using a HAAKE RotoVisco 1 (Thermo Scientific) apparatus equipped with an extended temperature cell for temperature control using a stainless-steel cone-and-plate (d = 60 mm, θ = 0.5°). The viscosity was measured at constant room temperature as a function of the shear rate in an upward sweep from 1 to 1000 s^−1^. The dynamic viscoelastic characterization of the solutions was determined by the frequency dependence (0.05–20 Hz) of the storage and loss moduli, G′(ω) and G″(ω), respectively, at 25 °C after inducing the gelation. Measurements were conducted with a rheometer TA AR2000 (TA instruments, New Castle, DE, USA) equipped with an extended temperature cell for temperature control and a stainless-steel parallel plate (d = 40 mm). The oscillatory shear experiments were performed at frequencies between 0.05 Hz and 20 Hz. The samples were allowed to equilibrate at room temperature for at least 20 min before the rheological test.

### 3.10. Nanoparticle Tracking Analysis (NTA)

Particle size was determined with a NanoSight NS300 device (Malvern Instruments, Malvern, UK) equipped with a red (642 nm) laser and an SCMOS camera. Data were collected and analyzed using NanoSight NTA 2.3 software. The samples were filtered using a 4 mm, 0.22 μm Millex syringe and diluted with DDW prior to analysis to achieve the desired particle concentration, between 10^7^ and 10^9^ total particles/mL. Temperature was maintained at 25 °C for the duration of the experiment, and a standard operating procedure (SOP) was created by using four captures of 60 s. The sample was advanced every minute, changing the particles in view to ensure a truly representative sample of particles. The samples were measured with a manual shutter and gain adjustment. Particle size was corrected for the viscosity of the continuous phase. The sample chamber was manually cleaned with MilliQ water and 10% (*v*/*v*) ethanol in water. Upon completion, no particles were visible in the sample chamber throughout the viewing window. All four measurements were averaged to obtain the mean, mode, and median particle sizes, together with an estimate of the number of particles.

## 4. Conclusions

We studied the effect of ultrasonication on the antiviral–physicochemical relationship of ἰ-carrageenan. The retention peaks in the FTIR spectra of ἰ-carrageenan and its ultrasonicated forms were similar, indicating that the chemical composition and its typical fingerprints were unchanged following ultrasonication. Ultrasonication for longer durations changed the structure of ἰ-carrageenan without considerable degradation; the polysaccharide became more hydrophilic (more hydroxyl groups were exposed to water), probably due to a reduction in the interactions between the polysaccharide chains. Following the ultrasonication, a significant reduction in viscosity was observed, and after 30 min of ultrasonication, the ἰ-carrageenan lost its gel structure. The ultrasonication also led to the formation of smaller nanoparticles, most of which were 100–300 nm in size. The low molecular weight ἰ-carrageenan fractions that were collected following 30 min of ultrasonication exhibited a significantly reduced antiviral activity, as compared with the 30 min-ultrasonicated ἰ-carrageenan and with the native ἰ-carrageenan. Since ultrasonication increased the antiviral bioactivity and reduced the viscosity of ἰ-carrageenan, we conclude that ultrasonicating the polysaccharide endows it with great potential to be used as an antiviral therapeutic agent. Our findings provide important insights into the relationship between the physicochemical properties and bioactivity of ἰ-carrageenan—and, potentially, also of other natural polymers—following ultrasonication, which may assist in the development of novel and more efficient therapeutic drugs against herpesviruses.

## Figures and Tables

**Table 1:** Power law index constants for ultrasonicated ἰ-carrageenan, and the coefficient of determination of the fitting.

Ultrasonication Duration (min)	n	R^2^
0	0.154	0.997
5	0.119	0.999
10	0.324	0.999
15	0.490	0.985
30	0.980	0.946

**Table 2:** Sugar content of native and 30 min-sonicated fractions of ἰ-carrageenan.

Fraction Size	% Sugar Content (Fraction/Total × 100)	
	Native	Sonicated
>100 kDa	99.27	90.20
10–100 kDa	0.60	8.80
3–10 kDa	0.08	0.49
<3 kDa	0.05	0.51

## Data Availability

Not applicable.
